# Effect of the COVID-19 Pandemic on Stimulant Use and Antiretroviral Therapy Adherence Among Men Who Have Sex With Men Living With HIV: Qualitative Focus Group Study

**DOI:** 10.2196/30897

**Published:** 2022-05-31

**Authors:** Mariya Petrova, Michael Miller-Perusse, Sabina Hirshfield, Adam Carrico, Keith Horvath

**Affiliations:** 1 Miller School of Medicine University of Miami Miami, FL United States; 2 San Diego State University San Diego, CA United States; 3 SUNY Downstate Health Sciences University Brooklyn, NY United States

**Keywords:** stimulants, HIV, ART, antiretroviral therapy, MSM, men who have sex with men, COVID-19, pandemic, therapy, drug use, virtual focus groups, virtual health, medication adherence

## Abstract

**Background:**

Evidence suggests that economic, social, and psychological circumstances brought about by the COVID-19 pandemic may have a serious impact on behavioral health. Men who have sex with men (MSM) are disproportionally impacted by HIV and stimulant use, the co-occurrence of which heightens HIV transmission risk and undermines nationwide treatment strategies as prevention efforts for ending the HIV epidemic. There is a paucity of information regarding the potential impact of the COVID-19 pandemic on the substance use and HIV medication adherence in this key vulnerable population—MSM who use stimulants and are living with HIV.

**Objective:**

The aim of this qualitative study was to identify ways in which the COVID-19 pandemic has affected stimulant use and antiretroviral therapy (ART) adherence among a sample of MSM living with HIV.

**Methods:**

Two focus groups were conducted in August 2020 via videoconferencing technology compliant with the Health Insurance Portability and Accountability Act. Potential participants from an established research participant registry at State University of New York Downstate Health Sciences University were invited and screened for study participation on the basis of inclusion criteria. A semistructured interview guide was followed. A general inductive approach was used to analyze the data. Findings in two general areas of interest, the impact of the COVID-19 pandemic on stimulant use and ART adherence, emerged directly from the raw data.

**Results:**

A total of 12 ethnically diverse participants over the age of 25 years took part in the study. Results were heterogeneous in terms of the effects of the pandemic on both stimulant use and ART adherence among MSM living with HIV. Some men indicated increased or sustained stimulant use and ART adherence, and others reported decreased stimulant use and ART adherence. Reasons for these behavioral changes ranged from concerns about their own health and that of their loved ones to challenges brought about by the lack of daily structure during the lockdown phase of the pandemic and emotion regulation difficulties.

**Conclusions:**

The COVID-19 pandemic has had a differential impact on stimulant use and ART medication adherence among MSM living with HIV. The reasons for behavioral change identified in this study may be salient intervention targets to support ART medication adherence and lower stimulant use among MSM in the aftermath of the of the COVID-19 pandemic, as well as beyond.

## Introduction

As the world encounters the most significant pandemic since 1918, caused by SARS-CoV-2, studies have begun investigating the direct and indirect impact of the pandemic on various health outcomes. Recent evidence suggests the myriad economic, social, and psychological challenges introduced by the pandemic and related prevention measures (eg, shelter-in-place orders) have been associated with serious behavioral health consequences, including increased substance use [1].

Gay, bisexual, and other men who have sex with men (MSM) in the United States face disproportionate rates of substance use and are specifically more likely to use stimulants (eg, methamphetamine, powder cocaine, and crack cocaine) than heterosexual men [2-4]. MSM are also disproportionately impacted by HIV, accounting for approximately 66% of annual diagnoses [5]. The co-occurrence of these conditions poses unique challenges: stimulant use among MSM living with HIV has been associated with lower antiretroviral therapy (ART) adherence, substantially elevated viral load, and amplified HIV transmission risk [6-12], thus undermining national treatment as prevention efforts for ending the HIV epidemic.

There is reason to believe that MSM who are living with HIV and use stimulants may be particularly at risk for negative health outcomes related to the COVID-19 pandemic [13]. However, there is a paucity of information regarding the impact of the pandemic on the health behaviors of this population. To address this knowledge gap, we sought to identify ways in which the COVID-19 pandemic has affected stimulant use and ART adherence in a sample of MSM living with HIV.

## Methods

### Methods Overview

We conducted 2 virtual focus groups with a total of 12 participants in August 2020. Potential participants were invited to screen for eligibility via emails sent out in July 2020 to a research participant registry at State University of New York Downstate Health Sciences University. Eligibility criteria for the study included identifying as a cisgender man, having had sex with another man in the past 12 months, currently living with HIV (self-reported diagnosis obtained by a health care provider), and currently taking ART. Furthermore, to be included in the study, participants had to indicate at least some difficulties with ART adherence in the past 30 days on the 3-item Wilson measure [14,15] and screen positive for moderate to severe stimulant use (ie, cocaine or amphetamine-type stimulants) on the abbreviated version of the Alcohol, Smoking, and Substance Involvement Screening Test [16]. Finally, all participants were required to be at least 18 years of age, speak English, and reside in the United States. Men who met the eligibility criteria, consented to take part in the study, and participated in one of the two focus groups received a US $50 Amazon gift card as compensation for their time.

### Procedures

Both focus groups were conducted remotely using Health Insurance Portability and Accountability Act–compliant videoconferencing technology. Participants and facilitators joined the virtual focus group with both video and audio, though only an audio recording was retained for review and transcription. The groups were facilitated by a clinical psychologist with expertise in conducting multiple research studies with MSM living with HIV who use stimulants. He introduced himself as a cisgender white gay man sharing his professional interests and position. The study coordinator, a white cisgender woman with a master’s degree in public health, was also present at each focus group call to provide technical assistance. The focus groups were conducted in English and a semistructured interview guide with suggested probes to elicit and clarify responses was followed. The participants were asked about the impact of the COVID-19 pandemic on their stimulant use and ART medication adherence.

### Analysis

A general inductive approach was used to analyze the data [17]. In unison with the approach, findings emerged directly from the raw data, even though the focus groups were conducted with broad areas of interest in mind. Both transcripts were read by a PhD-level prevention scientist and licensed mental health counselor with long-standing clinical and intervention development expertise in the field of substance use prevention. A comprehensive codebook was developed with areas of interest, codes, and subcodes based on the participants’ responses. The person who developed the codebook and a first-year PhD student in clinical psychology independently coded one of the interviews to assess the functionality of the codebook and to refine it.

The final codebook contained 8 codes and 28 subcodes. Each of the coders coded both focus transcripts using the final codebook. Initial agreement on the first transcript was 86%. The agreement rate on the following transcript was 80%. The two coders discussed and reached 100% consensus on all final codes. Changes and refinements of the codebook were made after coding each transcript. After coding all interviews, the 2 coders met to discuss the most salient and commonly endorsed codes across the 2 focus groups. These codes were then merged into higher-order themes within the broad areas of interest (categories) identified prior to conducting the interviews.

### Ethical Considerations

Study procedures were approved by the institutional review board of the University of Miami (20190578).

## Results

### Participants

Table 1 shows the participant demographic data. A total of 12 stimulant-using MSM living with HIV took part in the 2 focus groups (7 men in one group and 5 in the other). All participants were over 25 years of age: 3 (25%) men were in the 26-35–year age bracket, 3 (25%) were in the 36-45–year age bracket, and 6 (50%) were aged 46 years or older. In total, 3 (25%) participants identified as Black or African American, 6 (50%) identified as Latinx, and 3 (25%) identified as white. Only 1 (8%) participant reported an HIV diagnosis in the last 12 months, while all others (n=11, 92%) reported having been living with HIV for longer than 1 year. In total, 9 (75%) participants reported amphetamine use alone, 2 (17%) reported cocaine use alone, and 1 (8%) reported both amphetamine and cocaine use.

**Table 1:** Participant demographic data (N=12).

Category		Frequency, n (%)
**Sex**		
	Male	12 (100)
	Female	0 (0)
**Age (years)**		
	26-35	3 (25)
	36-45	3 (25)
	≥46	6 (50)
**Race and ethnicity**		
	Black or African American	3 (25)
	White	3 (25)
	Hispanic or Latinx	6 (50)

### Qualitative Themes

The results are shown in accordance with the distinct themes that emerged during the data analysis in the two categories of interest: ART adherence and substance use. Table 2 provides a summary of the results. Regarding the effects of the COVID-19 pandemic on stimulant use, two dominant themes emerged: increased or sustained versus decreased stimulant use. Higher versus lower ART adherence were the 2 categories that were identified during the discussions regarding ART adherence. Our analysis also sought to elucidate the participants’ reasons underlying the differential impact of the COVID-19 pandemic on stimulant use and ART medication adherence among MSM living with HIV.

**Table 2:** Qualitative results summary.

Themes		Subthemes	Subtheme examples
**Category: stimulant use**			
	**Perceived reasons for increased or sustained stimulant use (4, 33% participants)**		
		Avoidance of negative affect during the stay-at-home orders	“Ever since corona I pretty much just been getting high all the time… I get lonely. Normally I’d be at work for an extra eight hours.”
		Re-experiencing of enjoyable activities during the stay-at-home orders	“‘Oh, I miss the feeling’ and I’ll go back to it. So, since I’ve been home more, I feel like I have used [stimulants] more than I have in the couple months prior to that per se.”
		Simultaneous availability of free time and financial resources	“They [workers] were getting 2,000 dollars a month… it's unusual for me [to have] both free time and money coming in… I kind of got to the point from sort of the end of March until the end of May where I had never really been sort of a constant user but I turned into one.”
	**Perceived reasons for decreased stimulant use (4, 33% participants)**		
		Concern for the one’s own well-being	“I had time on my hands… but I really wanted to make sure I stayed in, and it was good, you know?”.
		Concern for others’ well-being	“Well, my case is because I have family so I don't want to put them at risk and, going out, it would be a risk for them more than for me.”
		Lack of opportunities for social interaction	“Less [stimulant use]… only because there's nothing to do once I've used.”
**Category: antiretroviral therapy adherence**			
	**Perceived reasons for decreased antiretroviral therapy adherence (3, 30% participants)**		
		Lack of life structure owing to the stay-at-home orders	“For the first month, month and a half, my adherence percentage went way down… I didn't know what day it was. It was very hard, although I was working from home certain times… it's like, ‘Did I take it, did I not take it?’ I don't remember.”
		Medication access	“I could only get a one-month supply at a time when it wasn't being mailed to me. I had to, you know, because of how it was being paid for, I had to actually physically go into the drugstore and then there was this whole thing around doing that safely, so… my adherence went down.”

### Category 1: Stimulant Use

Of the total number of focus group participants (n=12), 4 men endorsed increased stimulant use and 4 reported that their stimulant use had decreased as a result of the COVID-19 pandemic.

#### Theme 1: Perceived Reasons for Increased or Sustained Stimulant Use

Among men who reported an increase in stimulant use, our findings were varied: one participant resumed consistent stimulant use after 18 months of sobriety, one progressed from occasional to daily use, one expressed an elevation in the frequency of his occasional use, and one transitioned from social to a greater amount of solitary use. The most endorsed reason for increased or sustained stimulant use consistently stayed at home during the lockdown phase of the government’s response to the pandemic. Men reported three other reasons for their increased or sustained stimulant use during the stay-at-home orders: to avoid negative affect, to re-experience activities that were enjoyable or exciting, and the simultaneous availability of time and financial resources. One participant associated loneliness with spending more time inside, which he related to his recent relapse in stimulant use:

I was clean for about 18 months before that and then I lost my job due to corona… Ever since corona I pretty much just been getting high all the time… I get lonely. Normally I’d be at work for an extra eight hours.

Another participant explained the following:

I'll do it [use stimulants] pretty often, then I'll stop for a year or two, then it's like, ‘Oh, I miss the feeling’ and I’ll go back to it. So, since I’ve been home more, I feel like I have used [stimulants] more than I have in the couple months prior to that per se.

A third participant expressed that the combination of free time and the availability of unemployment or stimulus financial support during the pandemic may have facilitated his increased stimulant use:

They [workers] were getting 2,000 dollars a month… it's unusual for me [to have] both free time and money coming in… I kind of got to the point from sort of the end of March until the end of May where I had never really been sort of a constant user but I turned into one.

#### Theme 2: Perceived Reasons for Decreased Stimulant Use

Three main subthemes regarding reasons for participants’ decreased stimulant use during the COVID-19 pandemic emerged. The first subtheme was concern for the well-being of oneself and of others. One participant expressed the following:

When we were locked down, I really curtailed mine [stimulant use], because I would not let myself fuck up… I took it very seriously in terms of…sheltering in place.

Similar to the men who increased their stimulant use, those who curtailed their use experienced having a lot of free time. However, the participants who reported lower stimulant use attributed that to their strong desire to remain healthy:

I had time on my hands… but I really wanted to make sure I stayed in, and it was good, you know?

Participants were motivated to decrease their stimulant use owing to not only personal health concerns but also concern regarding the health of those around them. Two participants explained the following:

Well, my case is because I have family so I don't want to put them at risk and, going out, it would be a risk for them more than for me.

But now with COVID and I, like, I have a daughter and I have someone who lives with me actually recovering from their drug addiction, so I wouldn't do nothing serious [stimulants] at home.

The third major driving force behind curtailing one’s stimulant use was the lack of opportunities for social interaction:

Less [stimulant use]… only because there's nothing to do once I've used.”; “For me, the hard stuff [stimulants] will be socializing with my friends.

### Category 2: ART Adherence

Similar to its effect on stimulant use, the COVID-19 pandemic appeared to have had an impact on ART adherence of the men in our study: equal numbers of participants expressed increased (n=3) and decreased (n=3) adherence to their HIV medication.

#### Theme 1: Perceived Reasons for Decreased ART Adherence

In total, 2 of the 3 participants who reported lower medication adherence since the beginning of the pandemic expressed that the lack of structure in their lives due to the stay-at-home orders made it difficult to keep track of their medication intake. One expressed the following:

For the first month, month and a half, my adherence percentage went way down… I didn't know what day it was. It was very hard, although I was working from home certain times… it's like, ‘Did I take it, did I not take it?’ I don't remember.

One participant attributed his lower ART medication adherence to medication access:

I could only get a one-month supply at a time when it wasn't being mailed to me. I had to, you know, because of how it was being paid for; I had to actually physically go into the drugstore, and then there was this whole thing around doing that safely, so… my adherence went down.

#### Theme 2: Perceived Reasons for Increased ART Adherence

Two main subthemes emerged as perceived reasons for participants’ increased adherence to ART medication: curtailed stimulant use and self-compassion.

Two men attributed their increased adherence to ART medication to their curtailed stimulant use:

I've found that since COVID I've actually used less [stimulants]. Before I would always just use at home and I would find that I would miss my doses

ART medication

Well, for me when it comes to that, to that binge [stimulant use] like you call it, I'm usually out of the house… And then it's more of not having the medication accessible than forgetting… So then, as soon as I get in the house, I start again, but during COVID it hasn't been more than one day.

One of the participants, who expressed higher medication adherence since the beginning of the COVID-19 pandemic, spoke about concern regarding his well-being and understanding of his potentially increased susceptibility to COVID-19:

COVID has really made me want to stay on top of my medication simply because I figure I don’t need anything else. On top of that having a compromised immune system.

## Discussion

### Principal Findings

It is of particular importance to gain insight into the impact of the COVID-19 pandemic on stimulant use and ART adherence among MSM living with HIV who use stimulants, as such factors could undermine the clinical and public health benefits of HIV treatment as prevention [18]. This study sought to investigate how the COVID-19 pandemic has impacted stimulant use and HIV medication adherence among MSM. Our analysis revealed that the COVID-19 pandemic had different effects on stimulant use and ART adherence for men living with HIV who use stimulants. Of the men who discussed their experiences with stimulant use during the pandemic, half reported increased or sustained stimulant use and half disclosed that their stimulant use had decreased. Similarly, of the participants who expressed how the pandemic has impacted their medication adherence, half experienced improvements in their medication adherence and half reported that they were less likely to be fully compliant with their ART regimen.

### Improved ART Adherence and Lower Stimulant Use During the COVID-19 Pandemic

One of the most prominent perceived reasons for experiencing improved health outcomes (lower stimulant use or higher medication adherence) during the pandemic was men’s concern for their health. Participants who had lower stimulant use displayed a clear understanding that owing to their HIV diagnoses, they may be more susceptible to contracting COVID-19 and that staying at home (rather than engaging in stimulant use, which, for many people, is achieved in social settings) and taking their ART medication is what would keep them protected. This finding is consistent with the health belief model, which postulates that a person’s perception regarding a personal threat of an illness together with their belief in the effectiveness of a recommended health behavior will predict the likelihood that they will adopt the behavior [19]. These results suggest that during major health crises, such as the COVID-19 pandemic, supporting vulnerable populations (eg, MSM with co-occurring HIV and stimulant use) in understanding their potentially heightened disease susceptibility, along with providing clear directions for disease prevention (eg, wearing masks in public), may be an effective public health strategy for some persons.

Altruism was another prominent feature of the narrative regarding ways in which the COVID-19 pandemic has influenced our participants to lower their stimulant use. Altruism can be broadly defined as the practice of selfless concern for the well-being of others [20]. Men in our focus groups expressed that they curtailed their use because the behaviors associated with partying (going out and interacting with others) would expose their family members to the risk of contracting COVID-19 or because stimulant use at home may compromise the substance use recovery of people living in the same household.

Prior research has found that helping others is associated with better mental health [21] and significantly protects against engaging in serodiscordant condomless anal intercourse [22]. People who use drugs are among the most stigmatized and mistreated in the United States [23,24]. Nonetheless, our results suggest that some MSM living with HIV who use stimulants are motivated by altruism for the benefit of others’ health and will alter their own substance use to protect others. Thus, altruism may be an important intervention target for stimulant use reduction among MSM.

Our findings demonstrate that decreased opportunities for social interaction during the COVID-19 pandemic appeared to have reduced some participants’ stimulant use. Stimulant use has been consistently described as facilitative of seeking and engaging in risky sexual practices (eg, condomless sex and multiple sexual partners) [12,25-29] and as increasing sexual arousal, lowering sexual inhibitions, and increasing personal confidence [30-32]. One study on MSM living with HIV who use stimulants identified two themes regarding motivation: sexual enhancement and negative affect associated with an HIV-positive serostatus [33]. Although interview questions in this study did not investigate participants’ motivations for stimulant use, it is plausible that men who were more motivated to use stimulants for sexual enhancement may have curtailed their use because of decreased opportunities to meet sexual partners during the lockdown stages of the pandemic. Thus, identifying the motivations underlying stimulant use among MSM and addressing those motivations (eg, by shifting the social norms around them or by additional skill-building to address the specific need) may be a viable means of stimulant use prevention during the COVID-19 pandemic and beyond.

### Lower ART Adherence and Increased Stimulant Use During the COVID-19 Pandemic

The extended, unstructured time spent at home during the initial lockdown phase of the COVID-19 pandemic emerged as a dominant theme during the discussion regarding reasons for lower ART medication adherence. Our participants struggled with the lack of daily anchoring activities (eg, work schedule) and found it more difficult to take their HIV medication consistently. Thus, an important public health strategy during the COVID-19 pandemic and beyond may consist of mental health clinicians, medical staff, and other health personnel who interact with MSM on ART to assist patients with creating a regimen or utilizing a mobile reminder system to ensure timely medication uptake.

During the prolonged periods of time spent at home, our participants also reported using stimulants to avoid negative emotions (eg, loneliness) as well as to experience positive ones, both of which are aspects of impulsivity. In the literature, impulsivity is regarded as a multidimensional construct consisting of five facets: negative urgency (tendency to act rashly when experiencing negative affect), positive urgency (tendency to act rashly when experiencing positive affect), sensation seeking (tendency to enjoy and pursue exciting activities), lack of premeditation (not considering the consequences of an act before engaging in it), and lack of perseverance (difficulties remaining focused on a tedious task) [34,35]. Thus, it may be that some participants described negative urgency (to avoid negative feelings such as loneliness) and positive urgency (to experience positive feelings) as reasons for increased or continued stimulant use during the pandemic. These findings are well supported by studies that show a significant association between impulsivity and severity of drug use among cocaine and methamphetamine users [36,37]. Both our qualitative findings and prior data suggest that impulsivity may be an important intervention target for the prevention or reduction of stimulant use among vulnerable populations such as MSM living with HIV.

Finally, men in our study highlighted how the response of the larger social systems to the COVID-19 pandemic may have impacted their medication adherence and stimulant use. Having to pick up medication in person and not having a medication supply for more than 3 months were barriers to participants’ ART adherence. Our analysis also made evident the notion that while distributing financial support during a large public health crisis is certainly necessary, it is simply not sufficient. In addition to monetary support, many people with co-occurring health risks and conditions may need additional assistance (eg, to help them structure their budgets, create daily routines, or maintain stable psychosocial functioning) while in quarantine.

### Limitations

Our results should be interpreted in light of some limitations. The focus groups were conducted with a small sample of MSM living with HIV who use stimulants. Although Guest et al [38] have provided evidence that thematic saturation occurs within the first 12 interviews with purposefully sampled participants, it is essential to recognize that during focus groups, not all 12 participants voiced responses regarding their drug use and medication adherence. Thus, thematic saturation may have not been reached, and there is a need for larger qualitative studies on the topic. This study’s results serve as potential avenues for exploration in larger examinations of the impact of COVID-19 on stimulant use and ART adherence among more robust samples. Finally, we acknowledge that this qualitative study was not intended to be generalizable, but rather to gain an initial understanding of the impact of the COVID-19 pandemic on a specific group of people (MSM living with HIV with suboptimal ART adherence) and thus should not be interpreted as such.

### Conclusions

Our qualitative findings suggest that the COVID-19 pandemic is not having the same impact on all MSM who are living with HIV and are using stimulants. Indeed, our results show that different men may be responding differently to the pandemic in terms of stimulant use and ART adherence. This formative qualitative study identified some potentially salient intervention targets to support HIV medication adherence and lower stimulant use among MSM (eg, impulsivity, altruism, and motivation for stimulant use) during the COVID-19 pandemic and beyond. Importantly, the results from this study show nuanced reasons for adjusting their stimulant use and ART adherence patterns. The narrative of this study’s participants demonstrated that MSM living with HIV who use stimulants and have suboptimal compliance with ART medication practice self-compassion and altruism, modifying their health risk behaviors to strengthen their own health and to protect that of others. Finally, this study highlights the importance of easy and flexible access to medication, as well as the need to provide comprehensive support (eg, financial and psychosocial) to vulnerable populations.

## Abbreviations

ART: antiretroviral therapy
MSM: men who have sex with men
